# Two Fatty Liver Conditions Masquerading as Autoimmune Hepatitis

**DOI:** 10.1155/2021/8820350

**Published:** 2021-03-09

**Authors:** Jonathan David Hendrie, Mary Crouse Blumer, Hubert Henry Fenton, Gary Anthony Abrams

**Affiliations:** ^1^Prisma Health-Upstate, University of South Carolina School of Medicine-Greenville, Department of Medicine, 701 Grove Rd., Greenville, SC 29605, USA; ^2^Prisma Health-Upstate, Department of Pathology, 701 Grove Rd., Greenville, SC 29605, USA; ^3^Prisma Health-Upstate, University of South Carolina School of Medicine-Greenville Department of Medicine, Division of Gastroenterology & Liver Center, 890 W. Faris Rd., Suite 100, Greenville, SC 29605, USA

## Abstract

Acute fatty liver of pregnancy (AFLP) is a rare obstetric condition that classically presents in the third trimester or early postpartum period and can lead to liver failure and death. Only six second trimester AFLP cases have been reported in the English literature. We present the earliest case of AFLP at 15 weeks of gestation confounded by a high titer anti-nuclear antibody (ANA >1 : 1280) and concern for autoimmune hepatitis. Our patient had intrauterine fetal demise with prompt dilation and evacuation. Sepsis and multisystem organ failure ensued, and she was transferred to a liver transplant center where she expired without further intervention.

## 1. Introduction

Acute fatty liver of pregnancy (AFLP) is a rare and potentially fatal condition characterized by microvesicular steatosis and liver failure. The pathophysiology remains unclear, but research has implicated defective mitochondrial fatty acid oxidation as a contributing factor. AFLP typically presents in the third trimester or early postpartum period, but rare cases have been described in the second trimester [1–6]. Diagnosis is usually based on the Swansea criteria [7] in conjunction with serologic and biochemical testing although liver biopsy can be performed under certain circumstances. Treatment involves early diagnosis, prompt fetal delivery, and intensive supportive care with recovery expected over the ensuing days to weeks [8].

## 2. Case Report

A 41-year-old gravida 4 para 2 patient at 15 weeks and 5 days of gestation (based on ultrasound) presented to an outside hospital with decreased appetite and severe vomiting. She had WHO class III obesity (BMI 45.1 kg/m^2^) but was otherwise healthy. Initial laboratory tests showed a white blood cell count (WBC) 14 k/*µ*L, hemoglobin (Hgb) 13.7 g/dL, platelets (PLT) 354 k/*µ*L, normal blood smear, aspartate aminotransferase (AST) 354 IU/L, alanine aminotransferase (ALT) 574 IU/L, alkaline phosphatase (ALP) 125 IU/L, and total bilirubin (TB) 4.8 mg/dL. Abdominal ultrasound and magnetic resonance cholangiopancreatography showed hepatic steatosis, pericholecystic fluid, and gallbladder wall thickening. Further workup revealed a positive anti-nuclear antibody (ANA) with 1 : 640 titer but was otherwise negative for infectious, metabolic, or serologic etiologies. She was treated with methylprednisolone and antibiotics (ceftriaxone and metronidazole) for presumed hyperemesis gravidarum and acalculous cholecystitis, respectively. By the fifth hospital day, she felt greatly improved and tolerated food by mouth; laboratory results showed AST 91 IU/L, ALT 185 IU/L, ALP 127 IU/L, and TB 7.3 mg/dL. She was discharged with a steroid taper and antiemetics.

Three days later, she presented with vomiting, pruritus, and jaundice despite medication adherence. Laboratory testing revealed WBC 18.4 k/*µ*L, Hgb 12.1 g/dL, PLT 166 k/*µ*L, AST 51 IU/L, ALT 88 IU/L, ALP 191 IU/L, TB 15 mg/dL, total protein 6.8 mg/dL, albumin 2.6 mg/dL, serum creatinine 1.0 mg/dL, and international normalized ratio (INR) 2.2 mg/dL. She was transferred to our facility due to her advanced age, history of preterm delivery, and hepatic decompensation. She was alert and oriented with stable vital signs on admission. Complete blood count and comprehensive metabolic panel showed comparable values to those at her outpatient visit. Human immunodeficiency virus, hepatitis B surface antigen, total core antibody, e antigen, and hepatitis C surface antibody testing were nonreactive. Extensive viral and bacterial testing was negative. She denied fever, sick contacts, or recent travel outside of the country. The patient was not taking acetaminophen, amiodarone, antiretrovirals, methotrexate, piroxicam, tetracycline, tolmetin, valproate, or other sources of drug-induced liver injury. Repeat ANA was positive with a >1 : 1280 titer; additional autoantibodies and anticardiolipin were negative.

The patient was admitted to the hospitalist service and various consultants assisted with care. An abdominal ultrasound showed hepatic steatosis and an incidental absent fetal heart rate later confirmed by fetal ultrasound on day 2. Dilation and evacuation were performed on day 3. By this point, the differential diagnosis included AFLP, hemolysis with elevated liver enzymes and low platelets (HELLP), intrahepatic cholestasis of pregnancy (IHCP), and autoimmune hepatitis (AIH). Lack of cholestasis on imaging (ultrasound and MRCP) was inconsistent with IHCP. Preeclampsia, HELLP, and AFLP were considered unlikely based on gestational age, absence of hypertension, appropriate platelet count, and normal peripheral smear. If due to AFLP, the presumption was that it should improve postpartum. However, laboratory work on day 4 showed a rising ALP 226 IU/L and TB 20.5 mg/dL. Due to her positive ANA, she was empirically treated with methylprednisolone out of concern for AIH.

**Figure 1:**
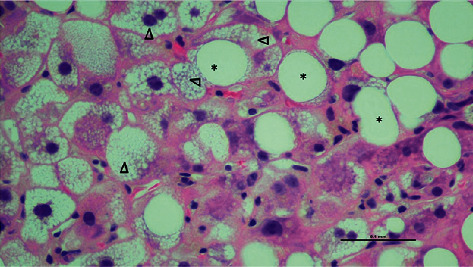
High power, hematoxylin and eosin (H&E) stained section of microvesicular (arrowheads) and macrovesicular (asterisk) steatosis.

**Figure 2:**
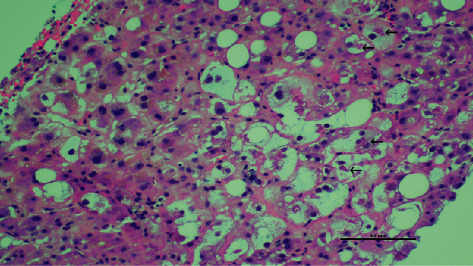
High power, hematoxylin and eosin (H&E) stained section showing cytoplasmic ballooning of hepatocytes with the presence of Mallory's Hyaline (arrows).

Liver biopsy was performed on day 5, which yielded inadequate tissue, but was suggestive of AFLP based on microvesicular steatosis. Acute renal failure, thrombocytopenia, and disseminated intravascular coagulation (DIC) developed over days 4–7, and a repeat biopsy on day 7 revealed significant microsteatosis, macrosteatosis, ballooning hepatocytes, and a mild inflammatory infiltrate without plasma cells, interface hepatitis, rosettes, or fibrosis (Figures 1 and 2). Several hours postbiopsy, the patient became encephalopathic, rapidly decompensated, and was transferred to a liver transplant center with multisystem organ failure. She expired before further intervention.

## 3. Discussion

There are varying reports on AFLP incidence, but most studies demonstrate a range between 1/7,000 and 1/20,000 births [9–11]. The pathophysiology is poorly defined, but dysfunction at the level of mitochondrial fatty acid (FA) oxidation is thought to play a role. Maternal-fetal FA levels are increased during pregnancy to support fetoplacental growth. One of the most well-supported theories of pathogenesis involves an inherited fetal deficiency of the long-chain 3-hydroxyacyl-coenzyme A dehydrogenase enzyme (LCHAD), which is part of a mitochondrial trifunctional protein (MTP) complex that catalyzes the *β*-oxidation of FA [12]. This autosomal recessive condition leads to the impairment of FA metabolism by the fetoplacental unit and subsequent buildup of toxic intermediate byproducts (i.e., long-chain metabolites) in the maternal circulation. Maternal LCHAD heterozygosity hinders the ability to clear toxins deleterious to hepatocytes [11, 13–15]. In a study of twelve women with a prior diagnosis of AFLP, eight were shown to be heterozygous for the LCHAD deficiency [16]. A separate cohort analysis found that 19% of AFLP cases had mutations in the MTP *α*-subunit responsible for LCHAD deficiency [13].

Most cases of AFLP occur in the third trimester or early postpartum period, but there are case reports as early as the second trimester with the earliest reported case at 18 weeks of gestation [1–6]. Clinically, AFLP leads to liver failure but is also associated with renal failure, coagulopathy, hypoglycemia, encephalopathy, and multisystem organ failure [17–19]—all of which occurred in the presented case. Presenting symptoms are often nonspecific and include fatigue, malaise, headache, nausea, vomiting, anorexia, and abdominal pain, which are obfuscated by the overlapping symptoms of mid-late pregnancy.

AFLP is a clinical diagnosis, and the Swansea criteria have been prospectively validated to aid diagnosis [7, 20, 21]. Based on these criteria, six or more of the following are required for diagnosis: vomiting, abdominal pain, polydipsia/polyuria, renal impairment, encephalopathy, coagulopathy, elevated ammonia, elevated urate, elevated bilirubin, elevated transaminases, hypoglycemia, leukocytosis, ascites or bright liver on ultrasound, and microvesicular steatosis on biopsy. A liver biopsy is rarely required for diagnosis and is often deferred due to the coagulopathic state of the patient [22] but can be justified in cases where liver function does not return to normal postpartum [23]. It is usually performed via trans-jugular approach and requires an adequate sample size of at least 2.5 centimeters with ten or more portal tracts [24–26].

Once diagnosed, management involves prompt fetal delivery and intensive supportive care with recovery expected over days to weeks [8]. Plasmapheresis initiated in the early postpartum period has demonstrated success in delaying or halting the progression of AFLP [27–30]. For patients with fulminant liver or multisystem organ failure despite the aforementioned treatments, auxiliary or orthotopic liver transplantation should be considered and has shown success [31–33]. A recent review of liver transplant outcomes in women of childbearing age showed that despite a decreased 5-year graft survival, patients transplanted for AFLP had comparable early and late survival outcomes compared to women transplanted for another reason [33].

Over the past few decades, maternal mortality in AFLP has decreased from 75–85% to 4–18% [8, 34–36]. Risk factors for adverse maternal outcomes include history of legal pregnancy termination, postpartum diagnosis, male fetus, intrauterine fetal demise, DIC, elevated serum creatinine, and prolonged prothrombin or activated partial thromboplastin time [17]. Fetal mortality is estimated at 20% [37], with negative outcomes correlated to fetal distress, prolonged partial thromboplastin time, fibrin degradation products, direct bilirubin, and decreased gestational age at delivery [3, 17]. Based on these factors, both the patient and her fetus were high risk for adverse events. Although there was diagnostic uncertainty due to the elevated ANA, our patient received the standard of care with prompt fetal delivery, biopsy, intensive supportive care, and transfer to a liver transplant center. In retrospect, plasmapheresis could have been considered but is not currently the standard of care. Whether the combination of AFLP and NAFLD portended this unfortunate outcome is unknown.

NAFLD is a general term for a group of conditions where fat accumulates in the liver of those who drink little to no alcohol. The most common condition under this umbrella term is fatty liver, which occurs when adipose accumulates in hepatocytes. Obesity is the primary risk factor for its development. In and of itself, fatty liver may not cause serious damage. However, a subgroup of patients may progress to develop nonalcoholic steatohepatitis (NASH), which is characterized by hepatocyte inflammation and liver scarring with the potential to cause cirrhosis.

Our case was challenging because AFLP has never been reported in the early second trimester and the presence of high ANA titers suggested an autoimmune etiology. To our knowledge, this is the first case that simultaneously supports two divergent fatty liver conditions: the uncommon AFLP and the very common NAFLD. AFLP seldom occurs in the second trimester, but our patient's progressive liver and renal failure, coagulopathy, hypoglycemia, encephalopathy, DIC, intrauterine fetal demise, and liver histology (characteristic diffuse microvesicular steatosis) support its diagnosis. To our knowledge, this is the earliest reported case of AFLP based on gestational age.

The concurrent diagnosis of NAFLD is supported by macrosteatosis in a patient with WHO class III obesity, as opposed to the microfat droplets of AFLP. Microvesicular steatosis is noted in 10% of NAFLD biopsies, but if present is associated with advanced fibrosis [38], which was absent in our patient. NASH is characterized by ballooning hepatocellular injury and inflammation as observed in our patient. Autoantibodies, even titers ≥1 : 640, are due to an epiphenomenon in NAFLD/NASH, and are not necessarily associated with advanced histologic features [39]. This explains the misinterpreted diagnosis of AIH, which also lacked support based on the absence of plasma cells, interface hepatitis, and rosettes.

There is an overlap between AFLP and HELLP syndrome, but there are enough factors that differentiate their diagnoses. This patient had a lactate dehydrogenase range of 586–660 U/L which is nonspecific with acute liver failure and does not necessarily point towards HELLP. The haptoglobin level was <8 mg/dL, but this was in the setting of DIC which can occur in either condition. HELLP is usually associated with elevated blood pressure, while AFLP is not [40], and the patient had no previous diagnosis of hypertension (admission blood pressure ranges: systolic 60–168 mmHg and diastolic 35–102 mmHg). Little can be drawn from these data during a prolonged hospitalization complicated by acute stress, D&E, volume overloaded requiring hemodialysis, and shock with vasopressor support. Second, acute renal failure is seen far more commonly in AFLP than HELLP (14–90% vs. 1.2–8.0%) [8–10, 19, 23, 40–43], and this patient was in fulminant renal failure requiring hemodialysis. Additional differentiating factors include hypoglycemia, encephalopathy, and coagulopathy which are described more frequently in AFLP due to hepatic insufficiency from steatosis and subsequent hepatocyte dysfunction [8–10, 19, 40–42, 44]. Throughout her hospitalization, INR ranged from 2.0 to 3.2 and she had seven hypoglycemic events (blood glucose < 70 mg/dL), even while tolerating a diet. Measurement of low antithrombin III levels has been described as useful in diagnosing AFLP but was not tested in this patient [44]. Lastly, the patient had biopsy-confirmed microvesicular steatosis which does not occur in HELLP.

In conclusion, we report that AFLP can occur in the early second trimester of pregnancy and should be in a clinician's differential diagnosis for acute liver failure regardless of gestational age. NAFLD is a common disorder associated with autoantibodies that can confound the diagnosis of liver diseases related to pregnancy.

## Data Availability

This case report involved a single patient whose data will remain restricted in accordance with the Health Insurance Portability and Accountability Act.
